# Incidence and clinical characteristics of zolbetuximab-induced nausea and vomiting in CLDN18.2-positive unresectable advanced or recurrent gastric cancer: a retrospective study

**DOI:** 10.1186/s40780-026-00569-z

**Published:** 2026-04-06

**Authors:** Yosuke Ando, Hiroki Banno, Satoe Yamaguchi, Akiko Ban, Kenji Katsuragawa, Ryo Sakurai, Yumiko Sato, Satoshi Nakamura, Toshinori Sasaki, Hirotaka Inoue, Satomi Kumazawa, Takuya Koyama, Saeko Harada, Etsuko Mishima, Hiroshi Matsuoka, Shigeki Yamada, Masayuki Miyazaki

**Affiliations:** 1 Department of Pharmacotherapeutics and Informatics, Fujita Health University School of Medicine, 1-98 Dengakugakubo, Kutsukake-cho, Toyoake, 470-1192 Japan; 2 Department of Pharmacy, Toyohashi Municipal Hospital, Toyohashi, Japan; 3 Department of Pharmacy, Kansai Rosai Hospital, Amagasaki, Japan; 4 Department of Pharmacy, Nishichita General Hospital, Tokai, Japan; 5 Department of Pharmacy, Toyota Memorial Hospital, Toyota, Japan; 6 Department of Pharmacy, NHO Nagoya Medical Center, Nagoya, Japan; 7 Department of Pharmacy, Nagoya City University West Medical Center, Nagoya, Japan; 8 Department of Pharmacy, Kasugai Municipal Hospital, Kasugai, Japan; 9 Department of Pharmacy, Mikawa Breast Cancer Clinic, Anjo, Japan; 10 Department of Pharmacy, NHO Toyohashi Medical Center, Toyohashi, Japan; 11 Department of Surgery, Fujita Health University School of Medicine, Toyoake, Japan; 12 Department of Hospital Pharmacy, Nagoya University Hospital, Nagoya, Japan

**Keywords:** Zolbetuximab, CINV, CLDN18.2, Gastric cancer

## Abstract

**Background:**

Zolbetuximab for CLDN18.2-positive advanced gastric cancer frequently causes chemotherapy-induced nausea and vomiting (CINV), particularly during the first infusion. Single-center studies in Japan have reported standardized infusion protocols and antiemetic strategies; however, these uniform approaches do not allow evaluation of how differences in infusion rate settings, antiemetic combinations, or patient backgrounds influence CINV. Using real-world data from multiple institutions with various clinical practices, the aim of this study was to clarify factors associated with CINV during the first zolbetuximab infusion, focusing on infusion rate, antiemetic use, and patient characteristics.

**Methods:**

This multicenter retrospective study included patients with CLDN18.2-positive unresectable or recurrent gastric cancer who received zolbetuximab between June 2024 and April 2025. Clinical characteristics, gastrectomy status, infusion parameters, antiemetic use, and CINV events during the first infusion were extracted from electronic medical records. CINV severity was assessed using CTCAE or patient-reported scales (NRS and Faces Scale), depending on institutional practice. Accordingly, CTCAE Grade ≥ 2, NRS ≥ 3, or Faces Scale ≥ 6 were defined as moderate-or-greater nausea. Patient backgrounds were summarized using descriptive statistics. Associations between CINV and age group, sex, gastrectomy status, antiemetic combinations, and infusion-rate categories were evaluated with exploratory analyses using Fisher’s exact or χ^2^ tests.

**Results:**

Forty-four patients were analyzed. CINV during the first zolbetuximab infusion was more frequent in patients without gastrectomy (38.7%) than in those with gastrectomy (7.7%). Younger age was associated with increased risk, whereas sex differences were minimal. Olanzapine use reduced the incidence of moderate-or-greater nausea or any-grade vomiting (18.2% vs 32.4%). CINV typically occurred when infusion rates reached approximately 200–250 mg/h, suggesting an increased risk when rates exceeded approximately 150 mg/h. Combined risk analysis showed that gastrectomy status had a stronger influence on CINV than age or sex, and olanzapine further reduced risk among non-gastrectomized patients.

**Conclusion:**

Infusion-phase CINV during the first zolbetuximab infusion was associated with both patient-related and administration-related factors in real-world practice. Quantitative evaluation of infusion-rate ranges and the influence of gastric mucosal status suggest that nausea severity may be partially modifiable through optimized infusion management and risk-adapted antiemetic strategies. These exploratory findings warrant prospective validation.

**Supplementary information:**

The online version contains supplementary material available at 10.1186/s40780-026-00569-z.

## Background

Zolbetuximab for patients with claudin 18.2 (CLDN18.2)–positive unresectable advanced or recurrent gastric cancer has shown a significant survival benefit compared with chemotherapy alone in the SPOTLIGHT [1] and GLOW [2] trials. However, a high incidence of chemotherapy-induced nausea and vomiting (CINV) was also reported. Nausea occurred in approximately 80% of patients and vomiting in about 60%, both at a higher frequency in the zolbetuximab-treated groups. CINV incidence was highest during the first and second infusions and was reported as an acute infusion-related adverse event occurring during drug administration. Furthermore, a higher infusion rate was associated with an increased risk of CINV, and interruption or reduction of the infusion rate was shown to alleviate symptoms. Patients without prior gastrectomy were more frequently affected, suggesting that gastric mucosal stimulation related to the target antigen may contribute to symptom development, given that CLDN18.2 is also expressed in a normal gastric mucosa [3]. In addition, CLDN18.2 is highly expressed in gastric epithelial cells, and preclinical studies using ferrets have shown that zolbetuximab-induced gastric mucosal inflammation may be associated with nausea and vomiting [4]. In both trials, antiemetic prophylaxis with a 5-hydroxytryptamine type 3 (5-HT3) receptor antagonist, a neurokinin-1 (NK-1) receptor antagonist, and dexamethasone was recommended; however, corticosteroids were sometimes withheld during the initial infusion owing to concerns regarding immunosuppression. Moreover, some patients received histamine H1 or H2 receptor antagonists, proton pump inhibitors (PPIs), or olanzapine, although the impact of these agents on emetogenicity has not been systematically evaluated. Collectively, these findings indicate that appropriate supportive care and careful infusion rate management are critical for preventing zolbetuximab-associated CINV. Nevertheless, the precise mechanisms underlying CINV and the optimal antiemetic regimen for zolbetuximab remain incompletely elucidated.

In Japan, a detailed real-world report on zolbetuximab-associated CINV was published by the Cancer Institute Hospital of Japanese Foundation for Cancer Research [5]. In that report, a prophylactic antiemetic protocol comprising a 5-HT3 receptor antagonist, an NK-1 receptor antagonist, and dexamethasone was administered at the initiation of zolbetuximab, with olanzapine added as needed. During the first infusion, nausea and vomiting were observed in 62.0% and 9.5% of patients, respectively, and symptoms consistently occurred during infusion-rate escalation from the initial rate of 75–250 mL/h. Temporary interruption of the infusion and administration of additional antiemetic agents resulted in symptom improvement in most cases, enabling all patients complete the first zolbetuximab infusion. Consistent with previous studies [1, 2], the incidence of CINV was lower among patients with a history of gastrectomy, suggesting an on-target/off-tumor effect related to gastric mucosal stimulation. These findings highlight the importance of multidisciplinary supportive care, including careful infusion-rate management and early intervention, for preventing and managing CINV during the initial zolbetuximab infusion.

In addition, real-world administration of zolbetuximab under a multidisciplinary safety management framework, designated as “Project VYLOY,” was reported by the Aichi Cancer Center Hospital [6]. In this report, all patients received a prophylactic antiemetic regimen consisting of a 5-HT3 receptor antagonist, an NK-1 receptor antagonist, and dexamethasone, with olanzapine added for refractory cases. Zolbetuximab was initiated at 100 mg/m^2^/h and gradually escalated in the absence of symptoms, resulting in nausea and vomiting during the first infusion, observed in 54.0% and 25.0% of patients, respectively. When Grade ≥ 2 symptoms occurred, the infusion was temporarily interrupted, additional antiemetics such as prochlorperazine or hydroxyzine were administered, and the infusion was resumed at a reduced rate. The interruption rate during the first infusion was 25.0%; nonetheless, it decreased to approximately 10% in subsequent cycles, allowing most patients to continue treatment safely with appropriate symptomatic management. These findings indicate that, in real-world clinical practice, standardized infusion-rate control combined with prompt antiemetic intervention is effective in improving tolerability to zolbetuximab during the induction phase.

More recently, updated stability data have shown that zolbetuximab remains stable for up to 12 h after preparation; however, prolonged infusion is not desirable from the perspectives of patient burden during hospitalization and nursing workload. Niigata Prefectural Shonai Hospital reported the safety and feasibility of outpatient zolbetuximab administration in a regional core hospital setting [7]. In this report, five patients with CLDN18.2-positive gastric cancer were managed in an outpatient setting using a multi-agent antiemetic regimen including olanzapine, fosnetupitant, palonosetron, and dexamethasone, together with a standardized protocol coordinated between the hospital pharmacy and community pharmacies. No Grade ≥ 3 adverse events were observed; nausea and vomiting were mild in all cases, and treatment adherence was maintained. In addition, a 10–15% reduction in medical costs relative to inpatient care was reported, suggesting more efficient use of healthcare resources. These findings indicate that outpatient administration of zolbetuximab is safe and feasible when adequate antiemetic prophylaxis and pharmacy–pharmacy collaboration are established.

However, all these previous reports were from single-center studies conducted under institution-specific protocols with standardized antiemetic regimens and infusion-rate settings. Consequently, the infusion-rate thresholds and escalation timing at which CINV is more likely to occur, as well as the most effective combinations of antiemetics, have not been sufficiently evaluated. Moreover, the impact of patient-related factors, such as gastrectomy status and age, remains unclear. Therefore, comprehensive analyses using multicenter data reflecting diverse clinical practices are required to better characterize the real-world determinants of CINV during zolbetuximab administration. Accordingly, the aim of this study was to clarify the effects of infusion rate, antiemetic use, and patient characteristics on the occurrence of nausea and vomiting during zolbetuximab treatment.

## Methods

### Patients

This multi-center retrospective cohort study analyzed patients with gastric cancer at eight hospitals, including Fujita Health University Hospital (Toyoake, Japan), Toyohashi Municipal Hospital (Toyohashi, Japan), Kansai Rosai Hospital (Amagasaki, Japan), Nishichita General Hospital (Tokai, Japan), Toyota Memorial Hospital (Toyota, Japan), NHO Nagoya Medical Center (Nagoya, Japan), Nagoya City University West Medical Center (Nagoya, Japan), and Kasugai Municipal Hospital (Kasugai, Japan), all of which are affiliated with the Cancer Section of the Aichi Prefectural Society of Hospital Pharmacists (Aichi Prefecture, Japan). Between June 2024 and April 2025, patients with CLDN18.2-positive unresectable advanced or recurrent gastric cancer who received zolbetuximab-containing regimens at eight participating institutions were retrospectively identified.

The exclusion criteria were as follows:

1. Lacking documented records of nausea and vomiting during zolbetuximab infusion.
2. Lacking treatment-related information, including infusion rate data for zolbetuximab.
3. Received phenothiazine tranquilizers, selective serotonin reuptake inhibitors (SSRIs), serotonin–norepinephrine reuptake inhibitors (SNRIs), serotonin–dopamine antagonists (SDAs), or multi-acting receptor-targeted antipsychotics (MARTAs) within 48 h before treatment initiation, except for olanzapine administered as premedication.
4. Received NK-1 receptor antagonists, 5-HT3 receptor antagonists, corticosteroids, or antidopaminergic agents within 48 h before treatment initiation for purposes other than premedication.
5. Newly initiated opioid analgesics within 48 h before treatment initiation.
6. Pre-existing nausea or vomiting at the start of zolbetuximab treatment.

### Study design and data collection

This study was conducted as a retrospective observational study using data from the electronic medical record. Collected variables included age, sex, height, body weight, body surface area, body mass index, history and extent of gastrectomy, and treatment regimen at therapy initiation. Data on zolbetuximab administration, including dose, infusion rate, and infusion duration, were collected. Information on antiemetic therapy was also reviewed, including the use (type and dosage) of NK-1 receptor antagonists, 5-HT3 receptor antagonists, corticosteroids, H1 receptor antagonists, olanzapine, D2 receptor antagonists, H2 receptor antagonists, and PPIs. In addition, the presence and severity of nausea, as well as the occurrence and frequency of vomiting during the first zolbetuximab infusion, were collected as outcome measures.

### Assessment

Patient- and treatment-related factors potentially associated with CINV were evaluated from multiple perspectives. Age was stratified into <50 and ≥50 years based on a previous report of a higher incidence of CINV in younger patients [8]. Sex was classified as male or female, considering prior reports indicating a higher risk in females [9]. Patients were categorized by the presence and extent of gastric resection, based on evidence suggesting a lower incidence of CINV, particularly among patients who underwent total gastrectomy [1, 2]. Infusion rates were standardized as mg/h and stratified into four categories: <150 mg/h, ≥150 to <250 mg/h, ≥250 to <450 mg/h, and ≥450 mg/h. The severity of nausea was assessed using the Common Terminology Criteria for Adverse Events (CTCAE) or patient-reported scales, depending on institutional practice. When patient-reported scales were used, the Numerical Rating Scale (NRS; 0–10) [10, 11] and the Faces Scale, which has six discrete levels (0, 2, 4, 6, 8, and 10) [12], were used. In symptom assessment research, nausea is frequently evaluated using a 0–10 NRS, and scores above 2.5–3 have been used to define more than mild or clinically meaningful nausea in previous clinical studies [13]. Furthermore, systematic reviews in cancer populations indicate that NRS scores around 3–4 represent the boundary between mild and moderate symptom intensity [14]. In addition, in the institutional clinical manual of the National Hospital Organization Hokkaido Cancer Center, Grade 2 nausea—defined according to the CTCAE, version 5.0—was mapped to Scale 3 on a six-level Faces Rating Scale (0, 1, 2, 3, 4, 5) based on expert consensus [15]. In that manual, Scale 3 represents a moderate symptom level within the six-point system and is interpreted as approximately corresponding to a score of 6 on the commonly used (0, 2, 4, 6, 8, 10) display format. Furthermore, in the participating institutions of this study, when NRS ≥ 3 or Faces ≥ 6 occurred, clinical management was implemented in a manner comparable to that for CTCAE Grade ≥ 2 nausea, including temporary interruption of zolbetuximab infusion and resumption at a reduced rate after symptom improvement. Accordingly, in this study, CTCAE Grade ≥ 2, NRS ≥ 3, or Faces Scale ≥ 6 were defined as moderate-or-greater nausea to standardize the assessment of nausea severity during infusion. This definition represents a pragmatic harmonization of clinician-reported and patient-reported measures rather than a validated direct conversion between scales. Vomiting was assessed according to CTCAE criteria.

### Statistical analysis

Patient characteristics were summarized using descriptive statistics, including means, medians, and standard deviations, as appropriate. The occurrence of CINV was summarized as frequencies and proportions by age, sex, gastrectomy status, and antiemetic use, and was explored using Fisher’s exact test or the chi-square test. Given the exploratory nature of this study, no predefined significance level was set; *p*-values were presented for descriptive purposes to identify trends.

For infusion rates at the first onset of CINV, medians and interquartile ranges were calculated, and the frequency and proportion of CINV were described using predefined infusion rate categories. For the exploratory analysis of the association between body surface area and nausea occurrence based on infusion rate, Kaplan–Meier curves were constructed, and between-group comparisons were made using the log-rank test. *p*-values were calculated for descriptive purposes. All statistical analyses were performed using EZR version 1.61 (Saitama Medical Center, Jichi Medical University, Saitama, Japan).

## Result

### Patient Background

Forty-four patients were included in this study. Being < 50 years, a factor potentially associated with increased emetogenic risk, and female sex accounted for 15.9% (*n* = 7) and 36.4% (*n* = 16) of CINV risk, respectively. The treatment regimens comprised mFOLFOX6 in 34.1% (*n* = 15) and CapeOX in 65.9% (*n* = 29) of patients. All patients received triple antiemetic prophylaxis with a 5-HT3 receptor antagonist, an NK-1 receptor antagonist, and a corticosteroid. In addition, olanzapine, antihistamines (H1/H2 blockers), PPIs, and gastric mucosal protective agents were administered at the discretion of each institution or treating physician (Table 1).

**Table 1:** Patient characteristics

	All patients (*n* = 44)
Age (<50) (n, ％)	7 (15.9)
Sex (Female) (n, ％)	16 (36.4)
BSA (m2), Median± SD	1.52 ± 0.18
BMI (kg/m2), Median± SD	18.2 ± 2.94
Regimen	
CapeOX (n, ％)	29 (65.9)
mFOLFOX6 (n, ％)	15 (34.1)
Gastrectomy (n, ％)	23 (52.3)
Total Gastrectomy (n, ％)	13 (29.5)
Distal gastrectomy (n, ％)	10 (22.7)
5-HT3 receptor antagonist	
Palonosetron (n, ％)	35 (79.5)
Granisetron (n, ％)	9 (20.5)
Dexamethasone	
3.3 mg (n, ％)	2 (4.5)
9.9 mg (n, ％)	42 (95.5)
NK1 receptor antagonist	
Fosnetupitant (n, ％)	33 (75.0)
Aprepitant, Fosaprepitant (n, %)	11 (25.0)
H1 receptor antagonist (n, ％)	29 (65.9)
H2 receptor antagonist (n, ％)	3(6.8)
Proton Pump Inhibitor (n, ％)	20 (45.5)
Gastroprotective agent (n, ％)	3 (6.8)
Olanzapine (n, ％)	10 (22.7)

BSA, body surface area; BMI, body mass index; SD, standard deviation

### Assessment methods for nausea severity

Across the eight participating institutions, nausea assessment methods varied according to institutional practice. One institution used the Numerical Rating Scale (NRS), one institution used the Faces Scale, and the remaining six institutions assessed nausea severity directly using CTCAE grading. At the patient level, cases 15–19 were evaluated using the Faces Scale, cases 35–44 were evaluated using the NRS, and all other cases were assessed using CTCAE grade. Regarding assessment timing, all institutions evaluated nausea at the initiation of zolbetuximab infusion, at each infusion-rate modification, and whenever patients reported symptoms during infusion. Nausea severity was assessed based on patient-reported symptoms and documented by trained healthcare professionals, including nurses or pharmacists, who interviewed patients and recorded the findings in the medical records.

### Nausea and vomiting during zolbetuximab infusion

CINV incidence during the first zolbetuximab infusion was compared using age, sex, history of gastrectomy, and antiemetic use (Table 2). CINV was observed across all subgroups; nevertheless, differences in incidence were observed based on patient characteristics. Regarding gastrectomy status, the incidence of moderate-or-greater nausea or any-grade vomiting was higher in patients without total gastrectomy (38.7%; 12 of 31) than in those who had undergone total gastrectomy (7.7%; 1 of 13). Patients younger than 50 years showed a higher incidence of CINV, whereas no apparent difference was observed between sexes. Regarding antiemetic use, the incidence of moderate-or-greater nausea or any-grade vomiting was lower among patients receiving olanzapine (18.2%; 2 of 11) than among those not receiving olanzapine (32.4%; 11 of 34). A lower incidence of vomiting was also observed in patients receiving H2 receptor antagonists. In contrast, patients receiving PPIs or gastric mucosal protective agents had a higher incidence of CINV, whereas no clear difference was observed between those using H1 receptor antagonists. Furthermore, analysis of the association between infusion rate and CINV showed that the median infusion rate at the first onset of any-grade nausea was 242.5 mg/h, whereas it was 250 mg/h for moderate-or-greater nausea and 245 mg/h for any-grade vomiting (Table 3).

**Table 2:** Incidence of CINV according to patient-related and treatment-related factors

	All patients (*n* = 44)							
	Nausea				Vomiting			
	All grade	P value	moderate-or-greater	P value	All grade	P value	≥Grade2	P value
All patients (%)	40.9 (18/44)		27.3 (12/44)		11.4 (5/44)		2.3 (1/44)	
Age (%)		0.42		0.37		1.00		0.16
≥50	37.8 (14/37)		27.3 (9/37)		10.8 (4/37)		0 (0/37)	
<50	57.1 (4/7)		42.9 (3/7)		14.3 (1/7)		14.3 (1/7)	
Sex (%)		1.00		1.00		1.00		1.00
Male	39.3 (11/28)		28.6 (8/28)		10.7 (3/28)		3.6 (1/28)	
Female	43.8 (7/16)		25.0 (4/16)		12.5 (2/16)		0 (0/16)	
Gastrectomy (%)		0.065		0.18		0.18		0.48
Yes	26.1 (6/23)		17.4 (4/23)		4.3 (1/23)		0 (0/23)	
Total Gastrectomy	15.4 (2/13)		7.7 (1/13)		0 (0/13)		0 (0/13)	
Distal gastrectomy	40.0 (4/10)		30.0 (3/10)		10.0 (1/10)		0 (0/10)	
Proximal gastrectomy	－		－		－		－	
No	57.1 (12/21)		38.1 (8/21)		19.0 (4/21)		4.8 (1/21)	
H1 receptor antagonist (%)		0.75		1.00		0.32		1.00
Yes	42.3 (11/26)		30.8 (8/26)		7.7 (2/26)		3.8 (1/26)	
No	38.9 (7/18)		22.2 (4/18)		16.7 (3/18)		0 (0/18)	
H2 receptor antagonist (%)		1.00		1.00		1.00		1.00
Yes	33.3 (1/3)		33.3 (1/3)		0 (0/3)		0 (0/3)	
No	41.5 (17/41)		26.8 (11/41)		12.2 (5/41)		2.4 (1/41)	
Proton Pump Inhibitor (%)		0.031		0.021		0.16		0.46
Yes	60.0 (12/20)		45.0 (9/20)		20.0 (4/20)		5.0 (1/20)	
No	25.0 (6/24)		12.5 (3/24)		4.2 (1/24)		0 (0/24)	
Gastroprotective agent (%)		0.56		0.18		1.00		1.00
Yes	66.7 (2/3)		66.7 (2/3)		0 (0/3)		0 (0/3)	
No	39.0 (16/41)		24.4 (10/41)		12.2 (5/41)		2.4 (1/41)	
Olanzapine (%)		1.00		0.70		0.57		1.00
Yes	40.0 (4/10)		20.0 (2/10)		0 (0/10)		0 (0/10)	
No	41.2 (14/34)		29.4 (10/34)		14.7 (5/34)		2.9 (1/34)

**Table 3:** Infusion rate and time to first CINV onset during zolbetuximab administration

	All patients (*n* = 44)			
	Nausea		Vomiting	
	All grade n = 18	moderate-or-greater n = 12	All grade n = 5	≥Grade2 n = 1
Time from infusion start to onset (min) (Median, IQR)	64 (59.3–89.3)	81 (59.8–109)	68 (63–79)	98 (NA)
Infusion rate(mg/h) (Median, IQR)	242.5 (177.3–390)	250 (199–370)	245 (177.3–300)	177.3 (NA)
Infusion rate				
<150 mg/h	22.2% (*n* = 4)	8.3% (*n* = 1)	20.0% (*n* = 1)	0% (*n* = 0)
≥150 mg/h and ＜250 mg/h	33.3% (*n* = 6)	41.7% (*n* = 5)	40.0% (*n* = 2)	100% (*n* = 1)
≥250 mg/h and ＜450 mg/h	38.9% (*n* = 7)	41.7% (*n* = 5)	40.0% (*n* = 2)	0% (*n* = 0)
≥450 mg/h	5.5% (*n* = 1)	8.3% (*n* = 1)	0% (*n* = 0)	0% (*n* = 0)

IQR, interquartile range; NA, not applicable

### Interaction of clinical risk factors and infusion characteristics in CINV

**Fig. 1:**
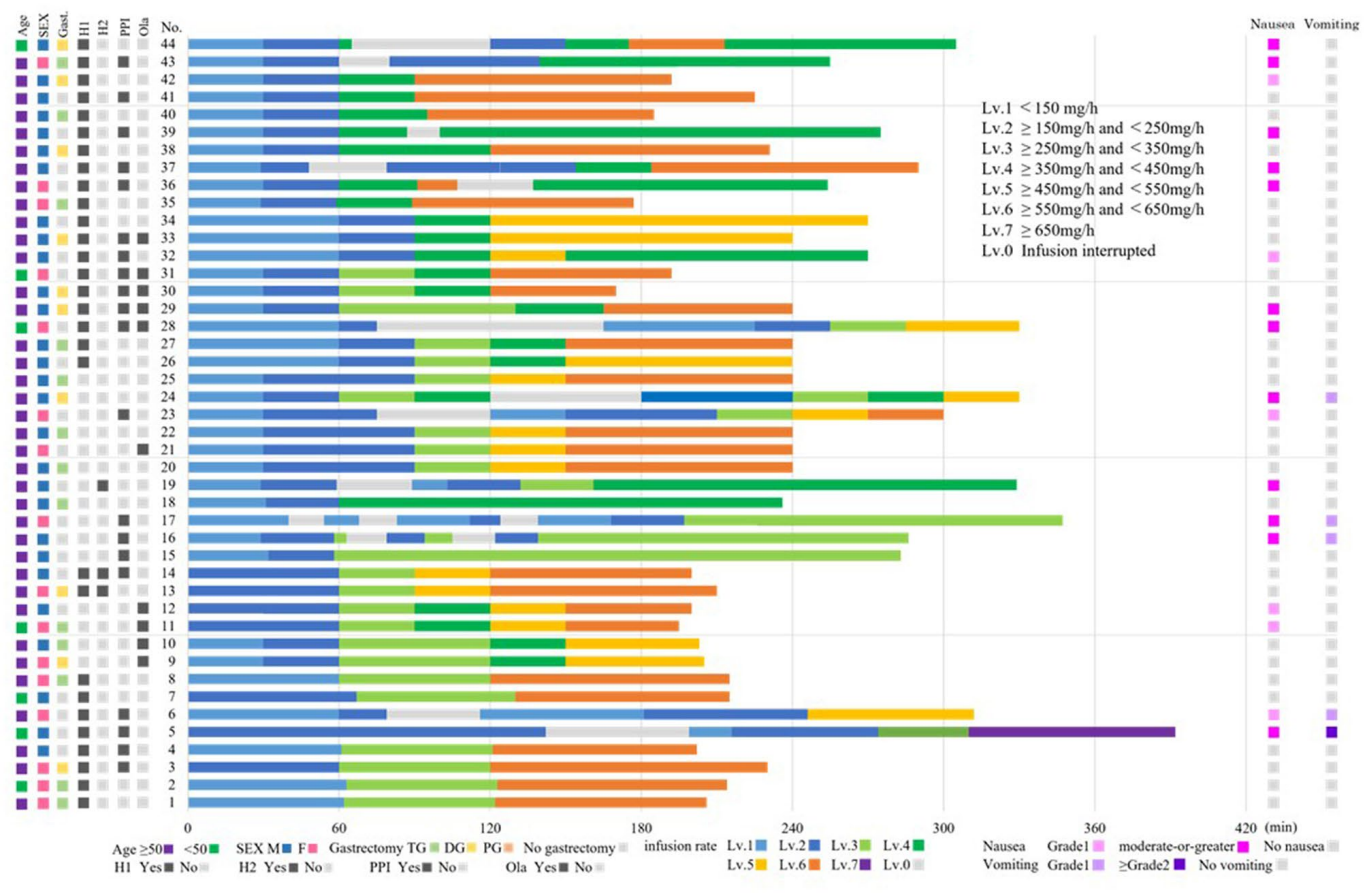
Infusion rate trajectory, CINV occurrence, and their association with patient characteristics during zolbetuximab treatment

Figure 1 summarizes CINV incidence according to patient characteristics and antiemetic therapy. Based on these findings, the interaction of established clinical risk factors and treatment-related variables was further explored. Subgroup analyses were conducted among younger (<50 years) or female patients. In this higher-risk population, CINV incidence varied according to gastrectomy status. Among patients with total gastrectomy, the incidence of any-grade nausea, moderate-or-greater nausea, and any-grade vomiting was 33.3%, 16.7, and 0%, respectively, compared with 53.8%, 38.5%, and 23.1% in those with non–total gastrectomy. In patients with Non–total gastrectomy, the impact of olanzapine was evaluated. The incidence of any-grade nausea, moderate-or-greater nausea, and any-grade vomiting was 37.5%, 25.0, and 0% in patients receiving olanzapine, compared with 56.5%, 39.1%, and 21.7% in those not receiving olanzapine (Table 4). In addition, infusion rates at the onset of CINV were analyzed according to baseline PPI use. Patients receiving PPIs tended to develop CINV at lower infusion rates than those not receiving PPIs. The median infusion rates at onset were 242 mg/h versus 300 mg/h for any-grade nausea, 200 mg/h versus 360 mg/h for moderate-or-greater nausea, and 211 mg/h versus 360 mg/h for any-grade vomiting in PPI users and non-users, respectively (Table 5).

**Table 4:** Comparison of CINV incidence in selected subgroups

	Nausea				Vomiting			
	All grade	P value	moderate-or-greater	P value	All grade	P value	≥Grade2	P value
Younger(<50) or female								
Total gastrectomy	33.3% (2/6)	0.63	16.7% (1/6)	0.61	0% (0/6)	0.52	0% (0/6)	1.00
Non–total gastrectomy	53.8% (7/13)		38.5% (5/13)		23.1% (3/13)		7.7% (1/13)	
Non–total gastrectomy								
Olanzapine (+)	37.5% (3/8)	0.43	25.0% (2/8)	0.68	0% (0/8)	0.29	0% (0/8)	1.00
Olanzapine off (-)	56.5% (13/23)		39.1% (9/23)		21.7% (5/23)		4.3% (1/23)

**Table 5:** Infusion rate at CINV onset according to baseline PPI use

	Infusion rate at CINV onset (mg/h)(median [IQR])							
	Nausea				Vomiting			
	All grade	P value	moderate-or-greater	P value	All grade	P value	≥Grade2	P value
PPI (+)	242 [194-210]	0.706	200 [196-300]	0.40	211 [158-259]	0.40	177 [177-177]	NA
PPI (-)	300 [210-390]		360 [280-380]					
							－	
					360 [360-360]			

## Discussion

While pivotal trials such as SPOTLIGHT and GLOW established the overall incidence of CINV associated with zolbetuximab and its association with gastrectomy status, detailed evaluation of clinically significant infusion-phase nausea under real-world infusion practices has not been sufficiently addressed. The present study focuses specifically on nausea occurring during infusion and examines its relationship with administration-related factors and patient characteristics. Unlike pivotal trials, which reported time to CINV onset without detailed infusion parameters, this study provides real-world data exploring how infusion management and supportive care variability may influence the development and clinical severity of infusion-phase CINV. In particular, quantification of infusion-rate ranges associated with nausea onset under routine clinical conditions has not been previously reported. These findings suggest that infusion-phase CINV may not be entirely inevitable but could be modifiable through optimized administration strategies.

The multicenter design allowed assessment of heterogeneous infusion practices and supportive care strategies under routine clinical conditions. Although all patients received guideline-recommended triple antiemetic prophylaxis, olanzapine was administered in only 22.7% of cases, indicating variability in implementation of quadruple antiemetic therapy in real-world practice. The use of adjunctive agents such as H1 and H2 receptor antagonists, PPIs, and gastric mucosal protective agents also differed across institutions. This heterogeneity provided an opportunity to explore how differences in antiemetic selection and infusion management may influence the incidence of infusion-phase CINV.

Consistent with previous reports, CINV incidence was lower in patients who had undergone total gastrectomy, supporting the role of CLDN18.2-expressing gastric mucosa in the emetogenic mechanism of zolbetuximab. However, because CINV still occurred in a subset of gastrectomized patients, additional mechanisms independent of direct gastric mucosal stimulation may be involved. Younger age was associated with higher CINV incidence, whereas sex-related differences were not observed, possibly owing to the small number of female patients included. These findings should therefore be interpreted cautiously.

Although the reduced incidence of CINV in patients with total gastrectomy is consistent with prior reports, the present study extends these findings by integrating gastrectomy status with infusion-rate analysis and antiemetic stratification. This integrated approach supports a mechanistic framework linking the residual presence of CLDN18.2-expressing gastric mucosa, drug exposure intensity, and the clinical severity of nausea during infusion.

In addition to patient-related factors, differences in antiemetic use were associated with CINV incidence. In this study, all patients who received olanzapine were administered prophylactic olanzapine on the evening before treatment (after dinner or at bedtime). These patients showed a lower incidence of moderate-or-greater nausea and any-grade vomiting. The efficacy of olanzapine for acute-phase CINV has been demonstrated in various highly emetogenic regimens [16, 17], including settings in which symptoms occur early after chemotherapy initiation [18]. Importantly, this finding should be interpreted cautiously in light of the distinct emetogenic mechanism of zolbetuximab. Preclinical studies have demonstrated that zolbetuximab induces retching and vomiting accompanied by gastric mucosal inflammation, suggesting that peripheral gastric mucosal irritation and vagal afferent activation play key roles in the initiation of emetic responses [4]. In such models, olanzapine did not significantly suppress emesis frequency, indicating that peripheral triggers may not be fully inhibited by centrally acting antiemetic agents. By contrast, the present analysis focused specifically on infusion-phase nausea during acute drug exposure, a patient-reported symptom occurring during the infusion period itself. This differs both from objectively measured vomiting frequency in animal models and from appetite loss assessed over multiple days in prior clinical studies [5], reflecting differences in symptom domain and temporal scope. Differences in assessment duration and clinical context may therefore partly explain the apparent discrepancy between studies. While peripheral mucosal stimulation may act as the initial trigger, the clinical severity of nausea is centrally integrated and modulated. Olanzapine antagonizes multiple neurotransmitter receptors involved in central emetic processing, including dopamine, serotonin (5-HT2/5-HT3), and histamine receptors, and may therefore attenuate the central perception and clinical severity of nausea rather than completely preventing emetic signaling. This interpretation is consistent with our results showing a reduction in clinically significant (moderate-or-greater) nausea rather than complete suppression of all CINV events. Nevertheless, given the retrospective design, limited sample size, and non-standardized antiemetic use across institutions, the observed association should not be interpreted as causal. Prophylactic olanzapine may have been preferentially administered to patients perceived to be at higher risk of CINV. Therefore, the present findings should be regarded as hypothesis-generating, suggesting that pre-treatment administration of olanzapine may help mitigate the clinical severity of infusion-phase nausea, which warrants further prospective validation under standardized antiemetic protocols.

Next, we examined the combined effects of patient-related background factors on CINV incidence. Among patients with at least one established risk factor—either younger age or female sex—CINV incidence appeared higher in those without total gastrectomy than in those who had undergone the procedure. This observation suggests that the influence of total gastrectomy may remain clinically relevant even when considering other established risk factors, supporting the possibility that the presence of gastric mucosa plays a role in the emetogenic mechanism of zolbetuximab. Furthermore, within this high-risk subgroup without total gastrectomy, CINV incidence appeared lower among patients who received olanzapine, suggesting a potential benefit in patients with preserved gastric mucosa. Nevertheless, patients receiving PPIs or gastric mucosal protective agents showed a higher incidence of CINV. These medications were primarily prescribed for pre-existing gastritis or duodenitis rather than for antiemetic prophylaxis, suggesting that underlying gastroduodenal mucosal injury or gastric vulnerability may have predisposed patients to zolbetuximab-induced CINV. However, this association should be interpreted cautiously and is unlikely to indicate a direct causal effect of PPI use itself. Rather, PPI use may serve as a clinical surrogate marker of pre-existing gastric symptoms or mucosal vulnerability. Given that zolbetuximab targets CLDN18.2 expressed on gastric mucosal cells, infusion-phase nausea may partly reflect local mucosal irritation and vagal afferent activation during drug exposure. Notably, in our dataset, the median infusion rate at the onset of moderate-or-greater nausea was lower in PPI users than in non-users (200 mg/h vs. 360 mg/h), suggesting that clinically significant nausea may occur even at lower exposure levels in patients with underlying gastric symptoms or mucosal sensitivity. This observation supports the hypothesis that the increased susceptibility to infusion-phase CINV in PPI users may be related to baseline gastric vulnerability rather than the pharmacological effect of PPIs themselves. From a clinical perspective, PPI users may therefore be considered a subgroup at higher risk of infusion-phase CINV, for whom individualized infusion management may be more appropriate than uniform escalation of antiemetic therapy. In particular, a lower initial infusion rate and more gradual rate escalation may reduce the intensity of acute mucosal stimulation and thereby attenuate infusion-phase nausea. Such infusion management directly addresses exposure-related symptoms and represents a practical and clinically applicable approach in routine practice.

Furthermore, infusion rate appears to substantially influence zolbetuximab-associated CINV. In the pooled analysis of the SPOTLIGHT and GLOW trials, the median time to first CINV onset was 50 min after infusion initiation; however, the corresponding infusion rates at that time were not specified. In this study, the median time to first CINV onset was 73 min, which is later than that reported in the pivotal trials. This difference may be attributable to the more cautious stepwise infusion-rate escalation adopted at some participating institutions. The median infusion rate at which CINV first occurred was 242.5–250 mg/h; furthermore, CINV incidence increased once infusion rates exceeded 150 mg/h. In the SPOTLIGHT and GLOW trials, infusion rates were adjusted based on body surface area (mg/h/m^2^), whereas absolute values (mg/h) without body surface area adjustment are used in real-world clinical practice. Consistent with this practice, infusion rates were expressed as mg/h in the present analysis. Using the study population’s median body surface area (1.52 m^2^) as the cutoff, patients were stratified into low and high-body-surface-area groups, and infusion rates at the first CINV onset were compared between groups. No clear difference was identified between the two groups, although a tendency toward CINV onset at lower infusion rates was observed in some patients with smaller body surface area (Additional Files 1 and 2). These findings suggest that infusion-rate settings based on absolute values (mg/h) without body-surface-area adjustment may be acceptable for zolbetuximab administration. However, given the limited sample size, these results should be interpreted with caution. Collectively, the present findings suggest that maintaining infusion rates up to approximately 150 mg/h may help reduce CINV incidence. Accordingly, for institutions currently initiating zolbetuximab at very low infusion rates, increasing the starting rate to approximately 150 mg/h may be a feasible option, provided that careful monitoring is ensured.

This study has some limitations. First, it was retrospective observational in design based on electronic medical record data, and the analysis focused exclusively on CINV occurring during the first zolbetuximab infusion. Therefore, CINV incidence during the post-infusion acute phase or the delayed phase across subsequent treatment cycles could not be evaluated. Second, owing to the limited sample size, multivariable analyses to identify independent risk factors for zolbetuximab-associated CINV were not feasible, and all findings should be interpreted as descriptive and exploratory. Accordingly, the reported *p*-values should not be interpreted as evidence of statistical significance but rather as indicators of trends intended for hypothesis generation. Third, nausea severity was assessed using a combination of clinician-reported CTCAE grading and patient-reported outcome measures (NRS and Faces Scale), and these were pragmatically harmonized into a unified definition of moderate-or-greater nausea. This approach may have introduced measurement variability and does not represent a validated conversion across assessment tools. Despite these limitations, the generally consistent trends observed across administration-related and patient-related factors provide a degree of support for the clinical relevance of the present findings.

## Conclusion

This multicenter real-world analysis suggests that infusion-phase CINV during the first zolbetuximab infusion is associated with both patient-related and administration-related factors. In particular, quantitative evaluation of infusion-rate ranges under routine clinical practice, including the observation of nausea onset at lower infusion rates in PPI users, provides novel insight into exposure-related susceptibility. The findings also suggest that prophylactic olanzapine may attenuate clinically significant nausea severity. These exploratory results support a risk-adapted approach to antiemetic prophylaxis and infusion-rate management and warrant prospective validation.

## Electronic supplementary material

Below is the link to the electronic supplementary material.

Supplementary Material 1

Supplementary Material 2

## Data Availability

The datasets used and/or analyzed during the current study are not publicly available due to ethical and confidentiality restrictions, but are available from the corresponding author on reasonable request. Access is subject to approval by the relevant ethics committees and compliance with confidentiality agreements.

## Abbreviations

CLDN18.2: Claudin 18.2
CINV: Chemotherapy-induced nausea and vomiting
NRS: Numerical Rating Scale
CTCAE: Common Terminology Criteria for Adverse Events
5-HT3: 5-hydroxytryptamine type 3
NK-1: Neurokinin-1
PPI: Proton pump inhibitor
SSRI: Selective serotonin reuptake inhibitor
SNRI: Serotonin–norepinephrine reuptake inhibitor
SDA: Serotonin–dopamine antagonist
MARTA: Multi-acting receptor-targeted antipsychotic
