# Supplementary material for: Incidence and clinical characteristics of zolbetuximab-induced nausea and vomiting in CLDN18.2-positive unresectable advanced or recurrent gastric cancer: a retrospective study
Source: J Pharm Health Care Sci. 2026 Apr 6;12:51. doi: 10.1186/s40780-026-00569-z (PMC13188557; doi:10.1186/s40780-026-00569-z)

Additional file 1 Impact of body surface area on infusion rate at first nausea onset during zolbetuximab administration

	BSA ＜1.52㎡	BSA ≥1.52㎡
Median, mg/h (95% CI)	NA (300-NA)	400 (240-NA)
P value	0.344	

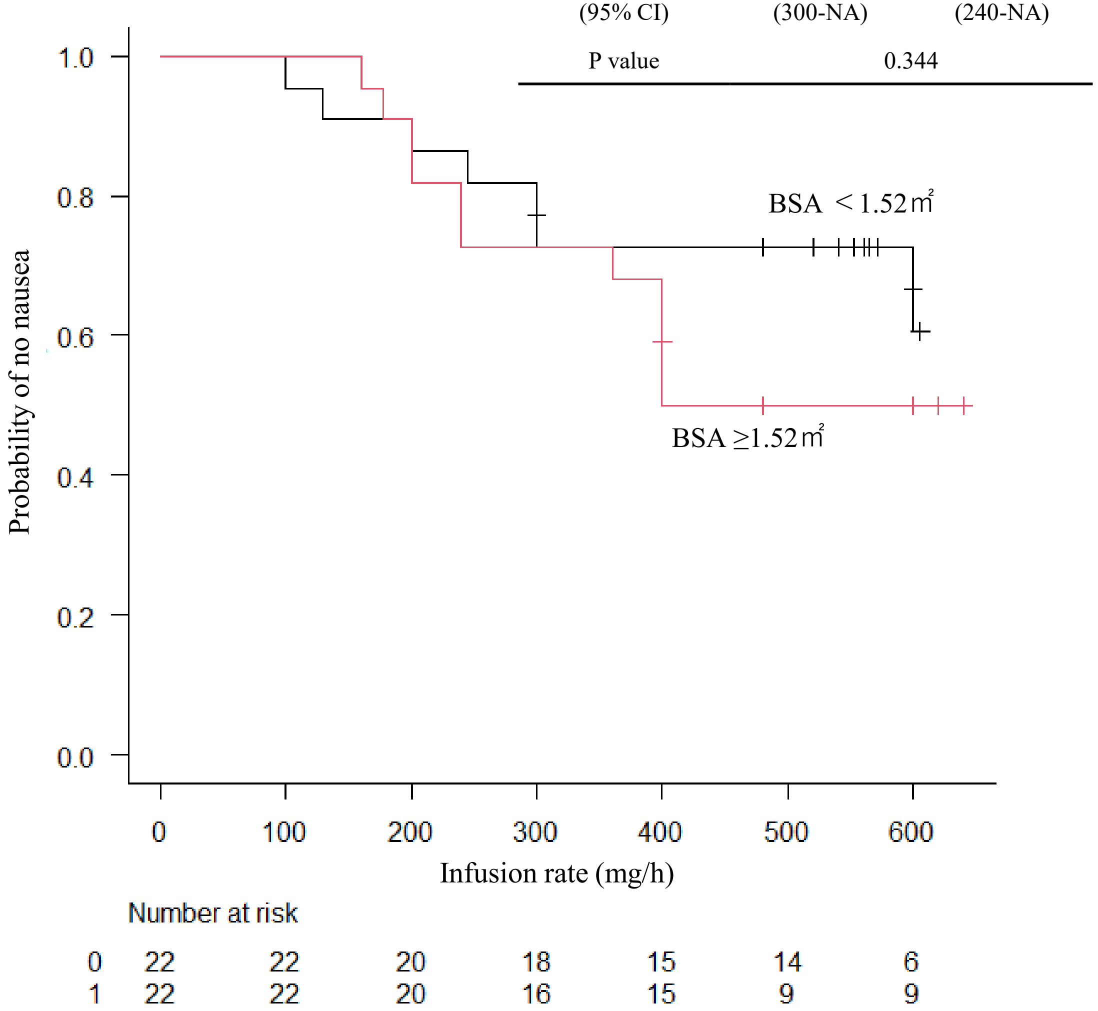

Additional file 2 Impact of body surface area on infusion rate at the first onset of moderate-or-greater nausea during zolbetuximab administration

	BSA ＜1.52㎡	BSA ≥1.52㎡
Median, mg/h (95% CI)	NA (600-NA)	NA (400-NA)
P value	0.844	

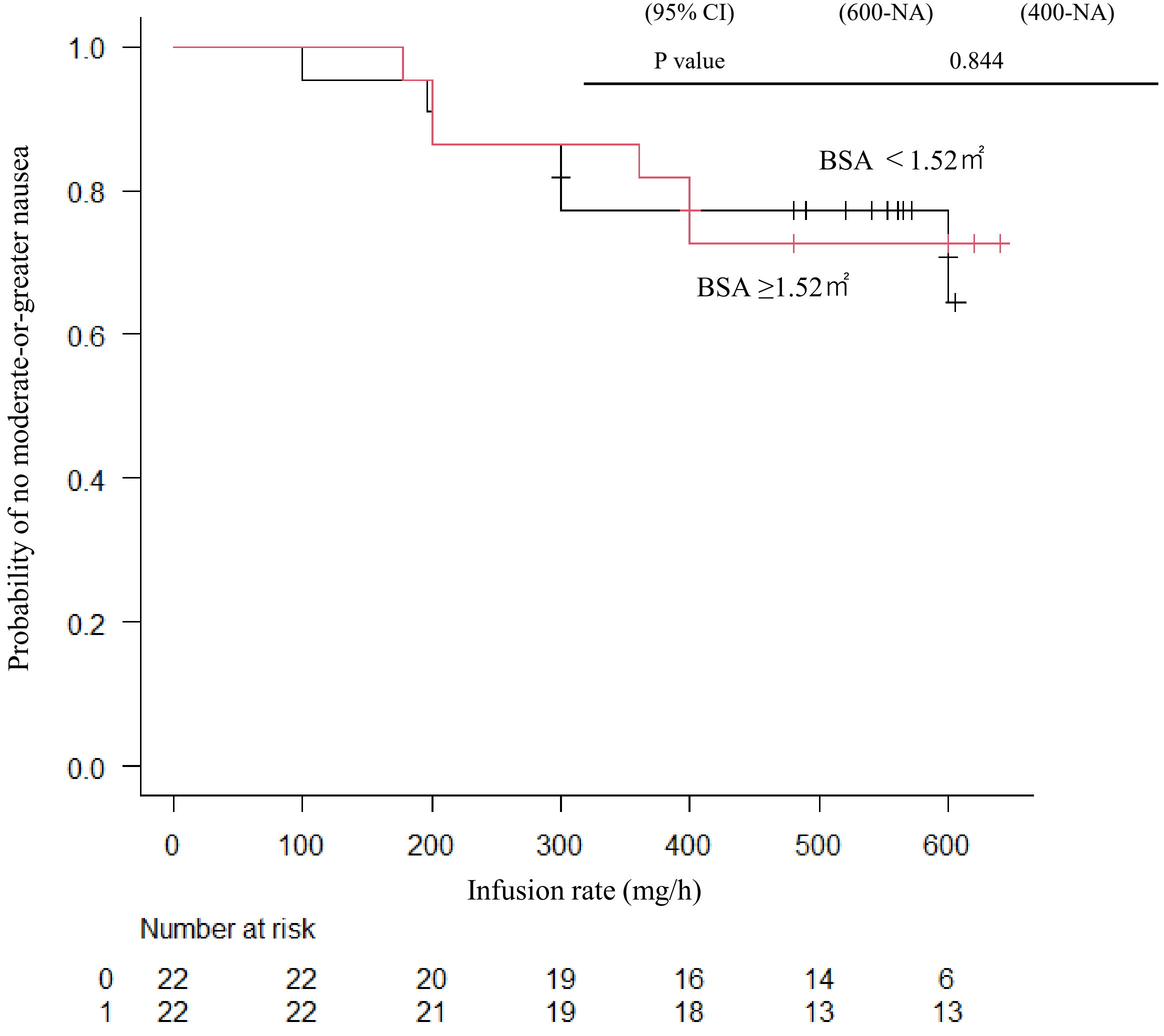

Supplement: Supplementary file 1 — Supplementary Material 1 [file 40780_2026_569_MOESM1_ESM.pdf]
