# Supplementary material for: Incidence and clinical characteristics of zolbetuximab-induced nausea and vomiting in CLDN18.2-positive unresectable advanced or recurrent gastric cancer: a retrospective study
Source: J Pharm Health Care Sci. 2026 Apr 6;12:51. doi: 10.1186/s40780-026-00569-z (PMC13188557; doi:10.1186/s40780-026-00569-z)
Supplement: Supplementary file 2 — Supplementary Material 2 [file 40780_2026_569_MOESM2_ESM.pptx]

## Slide 1
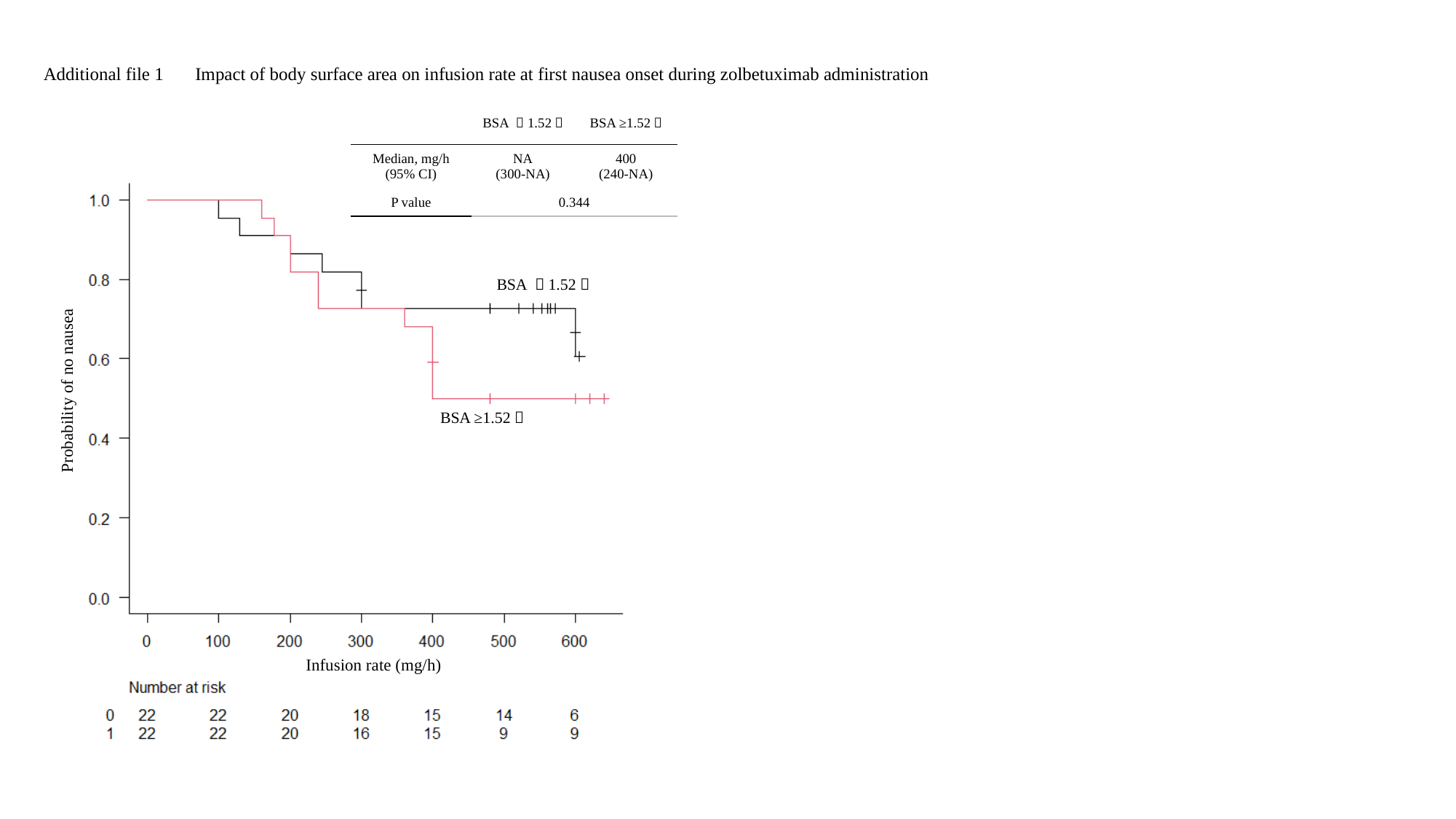

Additional file 1　 Impact of body surface area on infusion rate at first nausea onset during zolbetuximab administration
| | BSA ＜1.52㎡ | BSA ≥1.52㎡ |
| --- | --- | --- |
| Median, mg/h (95% CI) | NA (300-NA) | 400 (240-NA) |
| P value | 0.344 | |
BSA ＜1.52㎡
Probability of no nausea
BSA ≥1.52㎡
Infusion rate (mg/h)

## Slide 2
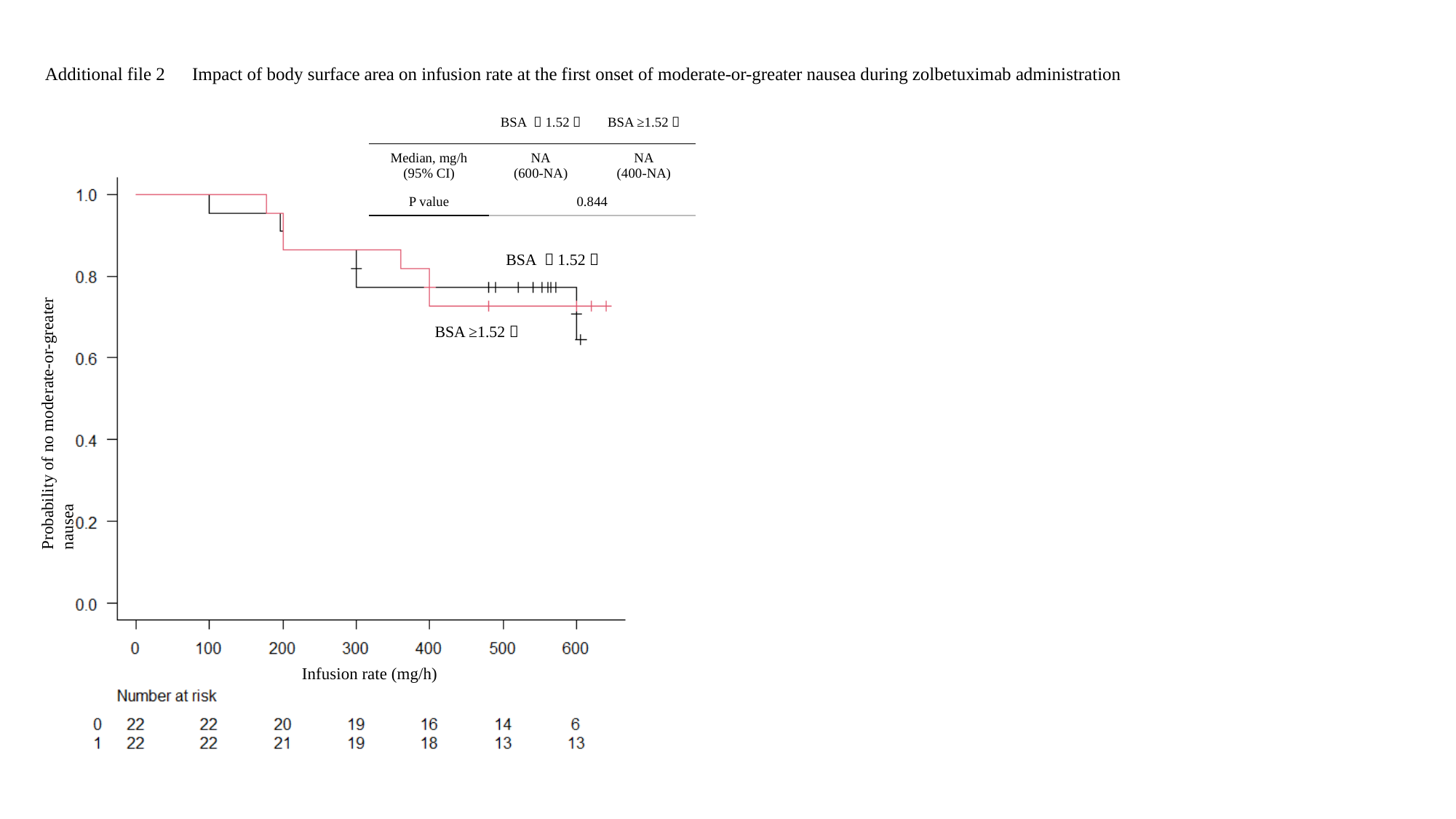

Additional file 2　Impact of body surface area on infusion rate at the first onset of moderate-or-greater nausea during zolbetuximab administration
| | BSA ＜1.52㎡ | BSA ≥1.52㎡ |
| --- | --- | --- |
| Median, mg/h (95% CI) | NA (600-NA) | NA (400-NA) |
| P value | 0.844 | |
BSA ＜1.52㎡
BSA ≥1.52㎡
Probability of no moderate-or-greater nausea
Infusion rate (mg/h)
